# The Potential of Albuminuria as a Biomarker of Diabetic Complications

**DOI:** 10.1007/s10557-020-07035-4

**Published:** 2020-07-17

**Authors:** Pappitha Raja, Alexander P. Maxwell, Derek P. Brazil

**Affiliations:** 1 Wellcome-Wolfson Institute for Experimental Medicine, Queen’s University Belfast, 97 Lisburn Road, Belfast, Northern Ireland BT9 7BL UK; 2 Nephrology Research, Centre for Public Health, Queen’s University of Belfast, Northern Ireland Regional Nephrology Unit, Belfast City Hospital, Belfast, Northern Ireland UK

**Keywords:** Albuminuria, Diabetic nephropathy, Atherosclerosis, Myocardial infarction, Heart failure, Peripheral arterial disease

## Abstract

Diabetes mellitus is a disease of dysregulated blood glucose homeostasis. The current pandemic of diabetes is a significant driver of patient morbidity and mortality, as well as a major challenge to healthcare systems worldwide. The global increase in the incidence of diabetes has prompted researchers to focus on the different pathogenic processes responsible for type 1 and type 2 diabetes. Similarly, increased morbidity due to diabetic complications has accelerated research to uncover pathological changes causing these secondary complications. Albuminuria, or protein in the urine, is a well-recognised biomarker and risk factor for renal and cardiovascular disease. Albuminuria is a mediator of pathological abnormalities in diabetes-associated conditions such as nephropathy and atherosclerosis. Clinical screening and diagnosis of diabetic nephropathy is chiefly based on the presence of albuminuria. Given the ease in measuring albuminuria, the potential of using albuminuria as a biomarker of cardiovascular diseases is gaining widespread interest. To assess the benefits of albuminuria as a biomarker, it is important to understand the association between albuminuria and cardiovascular disease. This review examines our current understanding of the pathophysiological mechanisms involved in both forms of diabetes, with specific focus on the link between albuminuria and specific vascular complications of diabetes.

## Introduction

Diabetes mellitus (DM) has long been recognised as a serious challenge to health, due to its ability to affect almost every organ in the human body. Diabetes is broadly classified into two subtypes: type 1 (T1DM) and type 2 (T2DM), with differing clinical phenotypes that require distinct therapeutic management. Both forms of diabetes are associated with an increased risk of cardiovascular complications that can have a significant impact on patient morbidity and mortality. Albuminuria is one of the most commonly assessed clinical parameters in diabetic patients. A number of simple dipstick testing methods are available in current practice to measure protein levels in the urine. Albumin is a tightly regulated component in the circulation, with the kidney playing an important role in maintaining albumin homeostasis. The role of albuminuria as a potential driver as well as a biomarker of diabetic complications has been increasingly recognised. Given the potential of albuminuria as a marker of diabetic vascular complications, this review will first summarise T1DM and T2DM, followed by the pathogenic processes linking albuminuria and cardiovascular complications of diabetes.

## Diabetes Mellitus

DM is a chronic endocrine condition that has reached pandemic levels worldwide. The disease is a growing public health concern, with 1 in 11 people worldwide affected by the condition [1]. Symptoms commonly associated with the onset of diabetes include increased urination (polyuria), increased thirst (polydipsia) and unintentional weight loss leading to increased appetite (polyphagia). Patients with diabetes have also reported slower wound healing, increased risk of acquiring infection, fatigue and blurred vision [2]. Clinically, DM is diagnosed in symptomatic patients when blood or urine sugar levels are elevated. A series of biochemical tests aid in confirming the diagnosis of diabetes in patients presenting with some of the symptoms listed above. In symptomatic patients, a fasting glucose concentration of ≥ 7 mmol/L (126 mg/dL) on two separate occasions is considered to be diagnostic for diabetes [3]. Haemoglobin (Hb) A1c has become widely used in clinical practice because it reflects long-term (3 months) glycaemic control [4–6]. A HbA1c reading of more than 48 mmol/mol (6.5%) supports the diagnosis of DM [3]. Based on clinical symptoms and age of onset, diabetes is broadly classified into T1DM and T2DM which account for approximately 10% and 90% of all diabetic cases respectively [2, 7]. Other forms of diabetes such as gestational diabetes and genetic forms of diabetes such as mature onset of diabetes in the young (MODY) also exist but will not be the focus of this review.

### Type 1 Diabetes

T1DM is caused by an auto-immune-mediated destruction of insulin-producing β-cells within the pancreatic islets of Langerhans [2]. Early studies have described diverse aetiologies for T1DM including genetic predisposition, environmental influences and immunological responses [8–12]. Many T1DM patients develop auto-antibodies against insulin and other antigens that contribute to the immune-mediated destruction of pancreatic β-cells. A number of cellular death pathways such as apoptosis and necrosis of pancreatic β-cells have been described in T1DM pathophysiology [13, 14]. Rojas et al. (2018) identified intrinsic and extrinsic apoptotic pathways which are regulated by the B cell lymphoma (Bcl)-2 and tumour necrosis factor (TNF) family of ligands respectively [15]. Experiments conducted by Thomas et al. have, however, questioned the significance of the TNF ligand, FAS, in mediating β-cell apoptosis as very few FAS producing cells were detected in T1DM mice models [16]. In contrast to apoptosis, necrotic and necroptotic processes initiate a cascade of immunological reactions in T1DM islets. Necroptosis or programmed necrosis is also facilitated by TNF signalling, which results in the synthesis of reactive oxygen species (ROS) [15]. In addition to this, calcium has been identified as a potent signalling modulator in necroptosis [17]. Genome-wide analysis has established interferon and tyrosine kinase 2 genes play a key role in eliciting apoptotic and necrotic pathways pancreatic β-cells [18].

### Type 2 Diabetes

The International Diabetes Federation estimates that there are currently 463 million people between the ages of 20 and 79 years living with diabetes [19]. Half of these patients (232 million) remain undiagnosed, and the number of diabetes patients is predicted to increase to 700 million by 2045 [19]. There is a strong genetic predisposition in both T1DM and T2DM pathogenesis. Mutations in *HLA* and *HNF1B* genes are strongly associated with the pathogenesis of T1DM and T2DM respectively [20, 21]. Currently, obesity is the strongest environmental risk factor associated with T2DM [22–24]. In the majority of individuals, obesity leads to insulin resistance and causes compensatory hyperplastic transformation of β-cells leading to hyperinsulinaemia that strives to overcome insulin resistance in peripheral tissues such as liver, muscle and fat. However, in T2DM patients, these early hypertrophic changes in pancreatic islets are overtaken by atrophic changes and a gradual loss of β-cells and insulin production. It is thought that obesity results in the de-differentiation of pancreatic α-cells into β-cells. Increased metabolic dysregulation induced by diabetes suppresses the transcription factor FOXO1. As a consequence, pancreatic β-cells undergo trans-differentiation into other types of pancreatic endocrine cells which leads to reduced pancreatic β-cell mass [24, 25].

Reduced insulin action in the liver, muscle and fat are key drivers of insulin resistance leading to hyperglycaemia in T2DM [26, 27]. Hyperglycaemia-induced metabolic dysregulation causes glucotoxicity which triggers pathogenic changes in T2DM [28]. Glucotoxicity increases reactive oxygen species (ROS) production resulting in glyceraldehyde-3-phosphate dehydrogenase (GAPDH) inhibition which subsequently decreases the antioxidant capabilities of β-cells [29]. Lack of glucose utilisation by adipose tissue promotes free fatty acid production which has been shown to induce programmed cell death of β-cells [30, 31]. Recently, the role of islet amyloid protein in β-cell death has been described [32, 33]. This polypeptide is released along with insulin in response to increased blood sugar levels. In diabetic conditions, there is increased secretion of the amyloid protein as a result of enhanced insulin secretion. Amyloid then accumulates within the endoplasmic reticulum of β-cells dysregulating cell cycle mediating transcription factors. As a result, apoptosis of β-cells is initiated within the pancreas, thereby reducing pancreatic β-cell mass, leading to insulin deficiency and hyperglycaemia [27–29].

## Diabetic Vascular Complications

The pathogenic changes described above can lead to dysregulated glucose homeostasis which can damage endothelial and other cell functions within the vasculature. These unregulated glucose levels are a major cause of chronic diabetic complications, which significantly increases mortality associated with DM [34–36]. Based on the organ and size of blood vessels affected, complications of DM are classified as macrovascular or microvascular. The heart, brain and peripheral vasculature are grouped as macrovascular complications of diabetes, which are major causes of death in people living with diabetes. For example, women living with diabetes have a 5-fold higher risk of myocardial infarction, an 8-fold higher risk of stroke and a 40-fold higher risk of requiring a foot amputation due to peripheral arterial disease (PAD) [37]. Cardiovascular conditions such as myocardial infarction (MI), heart failure and cardiomyopathies have been reported to be the major contributors of mortality in people living with diabetes [38]. Atherosclerosis is an inflammatory vascular condition which in the diabetic milieu accelerates cardiovascular and neurovascular diseases associated with macrovascular diabetic complications [39, 40].

The eye and the kidney are organs affected by diabetic microvascular complications. Diabetic eye disease or retinopathy is the leading cause of blindness worldwide [41]. Diabetic kidney disease, or nephropathy (DN), is the leading cause of end-stage renal disease (ESRD) in the working-age population [42]. Up to 50% of patients are diagnosed with diabetes with at least one diabetic complication evident (usually some degree of diabetic retinopathy). Both diabetic retinopathy and nephropathy are classified according to a series of stages, indicating the relative severity of the disease in each patient (Tables 1 and 2). These microvascular complications significantly impact the quality of life (e.g. visual loss) and reduce survival (e.g. ESRD). Between 25 and 40% of individuals with diabetes develop DN. Up to one-third of people with DN will progress to ESRD and diabetic kidney disease is now the leading cause of ESRD globally [42]. DN is diagnosed clinically when protein is detected in the urine and/or there is evidence of chronic deterioration in kidney function [3]. Given the poor prognosis for patients receiving chronic dialysis, together with the shortage of available donor kidneys for transplanation, a better understanding of the pathogenesis of DN and the role that albumin plays in this process is needed.

**Table 1:** Summary of clinical stages of diabetic nephropathy. Stages from G1 to G5 are shown, with associated estimated glomerular filtration rate (eGFR) and predicted renal function

Stage	eGFR (mL/min/1.73 m^2^)	Renal function
G1	≥ 90	Normal or high
G2	60–89	Mild reduction
G3a	45–59	Mild to moderate reduction
G3b	30–44	Moderate to severe reduction
G4	15–29	Severe reduction
G5	< 15	Kidney failure

**Table 2:** Summary of clinical classification of diabetic retinopathy (DR). Stages are classified in increasing order of severity from R0, R1 (background DR), R2 (Pre-proliferative) to R3 (Proliferative). The associated ophthalmoscopic findings are also indicated

Stage	Ophthalmoscopic findings
R0	No apparent lesions
R1 (background)	Microaneurysms
	Retinal haemorrhages
	Venous loops
	Exudate or cotton wool spots
R2 (pre-proliferative)	Venous beading
	Venous reduplication
	Intraretinal microvascular abnormality
	Blot haemorrhages
R3 (Proliferative)	New vessels on disc
	New vessels elsewhere
	Pre-retinal or vitreous haemorrhage
	Pre-retinal fibrosis
	Retinal detachment

## Albuminuria

The concept of measuring albumin in urine was first suggested by a German scientist, Hermann Senator, in the nineteenth century [43]. Albuminuria has been identified as the most sensitive marker for abnormal kidney function [44]. Stage I DN is characterised by microalbuminuria levels of 30–300-mg/24 h urine (Table 3). Measuring albumin levels from a 24-h urine sample has been considered the gold standard [2]. In 2012, The Kidney Disease: Improving Global Outcomes (KDIGO) organisation classified albuminuria as normal (< 30 mg/24 h), moderately increased/microalbuminuria (30–300 mg/24 h) and severely increased/macroalbuminuria (> 300 mg/24 h) (Table 3; [43]). A urine dipstick is the easiest and quickest way of detecting macroalbuminuria but it is a rather insensitive test for quantifying urine protein concentration. The urinary albumin-to-creatinine ratio (UACR) is convenient and equivalent to the gold standard technique for calculating ACR, with ratios < 3, 3–30 and > 30 mg/mmol being classified as normal, moderate and severe albuminuria respectively (Table 3; [2]). In addition to being a predictive marker of DN, albuminuria has also been found to be a useful prognostic marker for cardiovascular disease (CVD) in diabetic patients [45, 46]. To understand the intricate relationship between albuminuria and diabetic cardiovascular complications, it is first important to understand the function of albumin in the physiological setting.

**Table 3:** Classification of albuminuria in patients. Albuminuria is classified as A1 (normoalbuminuria), A2 (microalbuminuria) or A3 (macroalbuminuria). The relevant values for urinary albumin excretion rate (UAER), urinary albumin creatinine ratio (UACR) and albumin creatinine ratio (ACR), as well as the albuminuria severity banding, are indicated

Category	UAER (mg/24 h)	UACR (mg/mmol)	ACR (mg/g)	Severity	Classification
A1	< 30	< 3	< 30	Normal to mildly increased	Normoalbuminuria
A2	30–300	3–30	30–300	Moderately increased	Microalbuminuria
A3	> 300	> 30	> 300	Severely increased	Macroalbuminuria

## Albumin Homeostasis

Albumin is a small negatively charged protein synthesised mainly by the liver, contributing to 10% of overall protein production in the body. Albumin makes up ~ 75% of normal plasma colloid oncotic pressure and 50% of plasma protein content [47]. Albumin plays a key role in transporting molecules such as fatty acids, metals, bilirubin and hormones such as glucocorticoids in the plasma to their target cells [47]. Many commonly used drugs such as benzodiazepines, warfarin and cloxacillin bind to a number of different sites on albumin in plasma [48]. Albumin production is a highly regulated process modulated by various physiological and pathological conditions [49, 50]. Under normal conditions, levels of urinary albumin should be very low, with < 30 mg detectable in a 24-h urine sample (Table 3). A number of hormones, including glucocorticoids and insulin, are key regulators of albumin homeostasis [51]. In diabetes, the levels of these hormones are dysregulated, thereby leading to abnormal albumin metabolism. Breakdown of albumin occurs in most organs of the body, but mainly the muscle and skin [52]. The fractional catabolic rate of albumin is another important regulatory factor of albumin concentration in pathophysiologic states such as diabetes. In healthy individuals, renal tubular epithelial cells re-absorb the majority of albumin that is filtered by glomeruli and tubular cells can also degrade albumin via lysosomal proteolysis [53, 54]. In contrast, other authors report that albumin is protected from lysosomal degradation in tubular epithelial cells and is recycled intact to the circulation via tubular transcytosis [55].

## Albumin and the Kidney

Under normal conditions, the glomerulus, an intricate vascular structure in the kidney, limits the transport of albumin from blood to urine [54]. The glomerular filtration barrier is made up of glomerular endothelial cells on the blood vessel side, the glomerular basement membrane and podocytes with interdigitating foot processes on the urine side of the barrier [56]. Studies by Ryan and Karnovsky have identified the endothelial layer of the glomerular filtration apparatus to be the most effective barrier to albumin filtration [57, 58]. Multiple hypotheses have been postulated how this is achieved [57–61]. One of the first theories explored the size of the endothelial filtration pore as a preventative system for albumin filtration [62]. Gagliardini et al. carried out imaging of glomerular epithelial filtration slit using scanning electron microscopy in physiological and proteinuric conditions [63]. In this study, larger glomerular barrier pores were observed in mice with progressive renal fibrosis and diabetic nephropathy, thereby suggesting a positive correlation between albuminuria and dysregulation of glomerular pore size. Furthermore, Comper et al. examined the potential role of electrical charge as a barrier to albumin filtration [64]. Albumin and the glomerular vascular membrane are both negatively charged, suggesting that electrostatic repulsion of albumin molecules away from the vessel wall is an important component of barrier function [52]. This hypothesis was supported by a number of other reports suggesting that electrostatic repulsion was a major factor limiting the filtration of albumin from blood to urine [65–67]. In diseases such as diabetic nephropathy, the integrity of the glomerular filtration barrier is compromised by chronic hyperglycaemia, leading to increased albumin filtration into the urine (and detectable albuminuria).

**Fig. 1:**
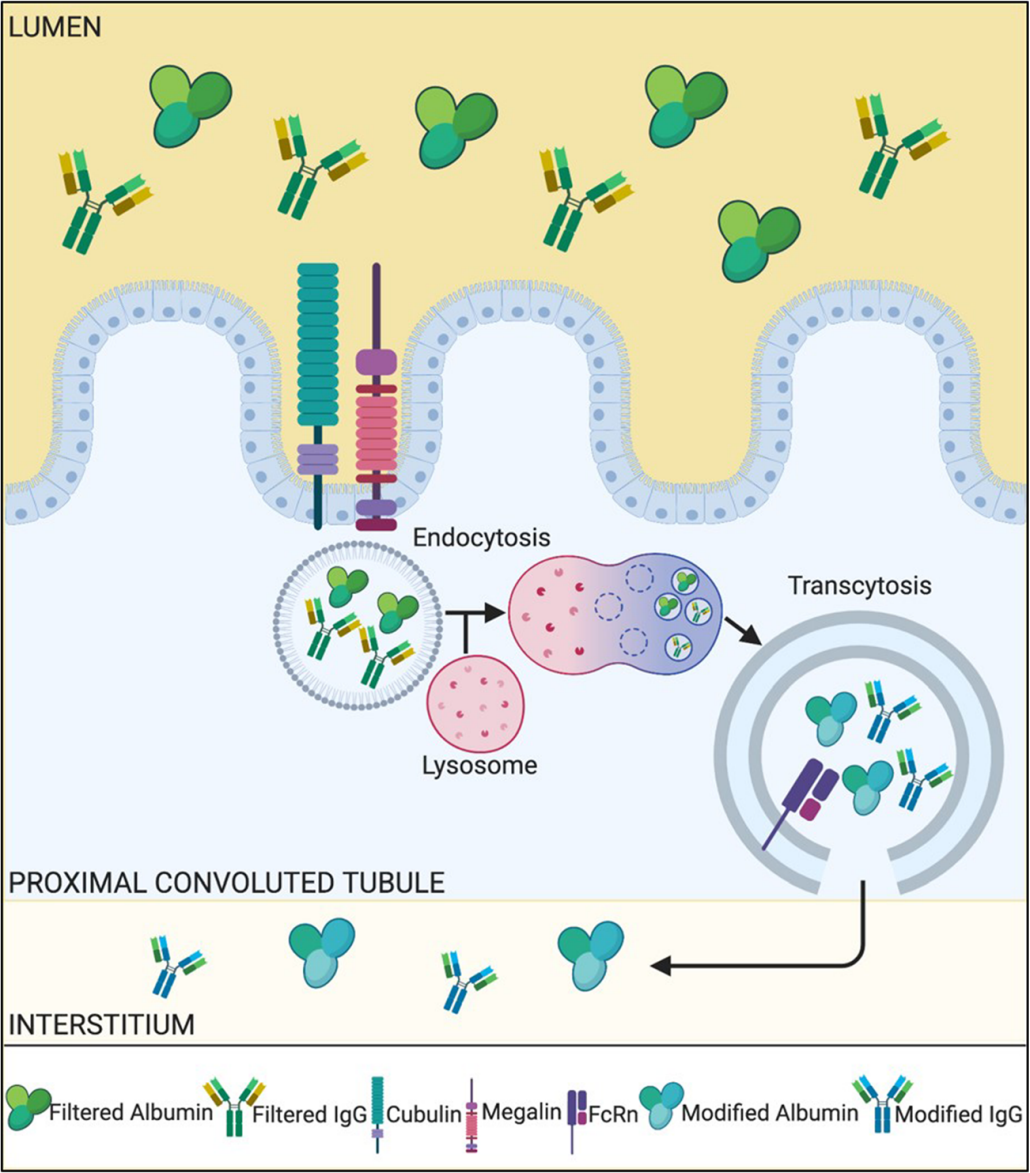
Schematic diagram showing how megalin and cubilin may contribute to albumin filtration from the blood vessels into the urine via proximal tubule epithelial cells. The key for each of the indicated molecules is shown at the base of the figure

The other nephron structure involved in regulating albumin excretion is the renal tubule (Fig. 1). In normal health, a small amount of albumin is filtered by glomeruli, and the majority of the filtered albumin is subsequently reabsorbed by the proximal and distal tubules [56]. A number of cellular transport processes, such as endocytosis and transcytosis, enable tubular epithelial cells to retrieve albumin from the glomerular filtrate [68–70]. In the proximal convoluted tubule, albumin binds to the megalin-cubilin receptor, resulting in endocytosis of the protein into vesicles which is either stored or degraded within the cytoplasm [69]. Many animal studies [69–73] have been carried out with results supporting this theory. An experimental study measured the levels of megalin and cubilin between diabetic and non-diabetic mice and identified the downregulation of these receptor proteins in diabetic mice [74]. Another study showed similar results and went on to demonstrate increased endocytosis and expression of renal megalin with insulin treatment in the diabetic group compared with the control group [75].

## Albuminuria and Diabetic Vascular Complications

### Albuminuria and Diabetic Nephropathy

Glomerulosclerosis and tubulointerstitial fibrosis are two major pathological hallmarks of DN [76]. Glomerulosclerosis is thought to occur as the initial insult in DN, followed by a progressive, irreversible fibrosis or scarring of the kidney tubules that occurs in parallel with the slow decline in renal function [76]. A variety of pathological mechanisms which contribute to diabetic kidney damage have been identified [42]. Injury to the glomerular filtration barrier leads to compromised filtration by the glomerulus which has been linked to the onset of renal fibrosis, potentially due to increased levels of albumin in the urinary filtrate [77]. Hyperglycaemia-induced haemodynamic dysregulation promotes increased production of endogenous vasodilators such as nitric oxide, insulin-like growth factor (IGF) and vascular endothelial growth factor (VEGF) [78]. Hyperglycaemia also promotes cellular oxidative stress resulting in the formation of advanced glycation end products (AGEs) [79]. These metabolic changes induce pathological changes in the glomerulus such as mesangial expansion, formation of Kimmelstiel-Wilson nodules and glomerular basement membrane (GBM) thickening [44, 76]. Damage to the glomerular basement membrane increases albumin excretion by increasing the pore size and disturbing the electrical homeostasis of the GBM.

Chronic hyperglycaemia is also associated with irreversible changes to renal tubular structure and function [75, 80, 81]. Transforming growth factor-beta (TGFβ) is an important cytokine driving the pathogenic changes in both diabetic glomerulus and kidney tubules [82]. TGFβ causes the expansion of glomerular mesangial cells, thickening of the GBM and loss of filtration barrier integrity [83]. TGFβ is also implicated in tubulointerstitial fibrosis, where activated myofibroblasts generated from vascular pericytes, tubular epithelial cells and resident fibroblasts secrete extracellular matrix proteins such as collagen IV and fibronectin that contribute to scar formation [84–86].

Recent experiments performed by Mori et al. analysed albumin excretion in diabetic *megalin* gene knockout mice [87]. A significant increase in albumin excretion was detected in diabetic *megalin* knockout mice compared with diabetic wild type [87], suggesting that lower megalin expression reduced the capacity of the kidney tubular epithelial cells to sequester albumin in the kidney filtrate. The potential role of megalin in potentiating TGFβ-induced epithelial-mesenchymal transition (EMT) has also been studied. TGFβ was used to stimulate EMT in epithelial renal cells and reduced levels of megalin were detected in these cells and these changes were reversed when the cells were treated with a TGFβ inhibitor [88, 89]. A meta-analysis of data from genome-wide association studies (GWAS) has identified a mutation in the cubilin-encoding gene *CUBN* that is associated with microalbuminuria in European and African populations [90].

Additionally, a post hoc analysis of data from the Diabetes Control and Complications Trial (DCCT) and Epidemiology of Diabetes Interventions and Complications (EDIC) study also confirmed an association between *CUBN* mutations and an increased risk of developing microalbuminuria [91, 92]. Increased cubilin expression was also observed in T1DM mice with cubilin mRNA levels negatively correlated with albuminuria [93]. These and other reports highlight the significance of the megalin-cubilin receptor pathway in tubular cell albumin uptake and the pathogenesis of diabetic kidney disease [72, 73, 78, 87, 94].

### Albuminuria and Atherosclerosis

The causative link between albuminuria and CVD has been extensively studied, yet the pathophysiological mechanism has not been clearly identified [95–98]. The most commonly accepted theory is the STENO hypothesis, which states that glomerular dysfunction is reflective of extensive vascular damage throughout the body [99]. In diabetes, processes such as endothelial dysfunction and chronic inflammation lead to the development of angiopathies in many organs. Microalbuminuria has been associated with an increased risk of developing subclinical atherosclerosis in the Mexican population, with another study reporting the same in elderly patients living with diabetes [100, 101]. The intimal-media thickness (IMT) is an early indicator of developing carotid atherosclerosis [102]. Zhang et al. assessed IMT using high-frequency ultrasonography and reported a strong correlation between microalbuminuria and IMT [103]. Multiple studies have examined the link between inflammatory cytokines and atherosclerosis accelerated by diabetes [104–106]. Cytokines such as interleukin-6 (IL-6) and tumour necrosis factor alpha (TNFα), along with acute-phase proteins like C-reactive protein (CRP), were shown to promote inflammatory processes within the vasculature leading to endothelial damage and dysfunction [107–110]. Despite strong evidence supporting the link between microalbuminuria and diabetes-induced atherosclerosis, recent evidence has identified microalbuminuria to be an independent risk factor for atherosclerosis even in the absence of diabetes [111, 112]. A cross-sectional study performed by Kimura et al. studied 1756 non-diabetic men with normal estimated glomerular filtration rate (eGFR), and the results indicated that patients with high-normal albuminuria had a significantly higher risk of IMT and developing atherosclerotic plaques [113]. A recent gene analysis study has identified mutations in the *APOL1* gene to be associated with this outcome, but further studies are needed to validate this finding [114].

### Albuminuria and Myocardial Infarction

Myocardial infarction (MI) is a cardiovascular condition which is commonly associated with microalbuminuria. Multiple clinical trials targeting different population groups have concluded that increased albumin excretion is associated with an increased risk of MI [106, 115–118]. Secondary analysis of data from the TRACER study has reported microalbuminuria was associated with an increased risk of MI and cardiovascular mortality [118]. Another study of Mexican patients with diabetes also identified a higher number of major adverse cardiovascular events associated with increased albuminuria levels [117]. These findings were further supported by the REGARDS study which examined persons with diabetes with and without coronary heart disease (CHD). The results concluded that higher urinary albumin-to-creatinine ratio was associated with greater risk of incident of heart disease in black versus white patients > 45 years in the USA [119]. These conclusions suggest that albuminuria is a strong biomarker for CHD development and mortality. The underlying pathogenic mechanisms are similar to those described for atherosclerosis, with aberrant endothelial signalling and low-grade vascular inflammation within coronary arteries being key drivers of MI in people with diabetes. Therefore, control of albuminuria clearly has the potential to improve the outcomes of patients at risk from MI and other cardiovascular diseases.

### Albuminuria and Heart Failure

Clinical studies have identified increased urinary albumin excretion among heart failure patients [118, 120–123]. A prospective study carried out in a cohort of patients with well-characterised heart failure and preserved ejection fraction showed increased urinary albumin excretion was associated with enhanced remodelling of the right and left ventricles [124]. During follow-up of this cohort, raised urinary albumin excretion was also associated with systolic dysfunction (assessed by echocardiography) [125]. Post hoc analysis of the SAVOR-TIMI 53 trial cohort also identified increased hospitalisation for heart failure among patients with microalbuminuria [126]. Another study recruited 100 asymptomatic people living with diabetes and compared their cardiac extracellular volume fraction with urinary microalbumin levels. A strong positive association was observed between these two measurements, suggesting diffuse cardiac fibrosis is the underlying pathogenic mechanism involved in heart failure development among patients with persistent microalbuminuria. The glomerulus is one of the first vascular structures to be affected by diabetes resulting in microalbuminuria. As stated previously, glomerular damage is considered to be indicative of a more widespread vascular injury including the heart.

Though a direct correlation between albuminuria and cardiac fibrosis has not been established, a number of clinical trials have identified existing diabetic medications which may reduce albuminuria and potentially attenuate fibrosis [127–131]. Sodium-glucose transport protein 2 (SGLT2) inhibitors are widely used medications for diabetes management which also has been shown to reverse microalbuminuria. Early studies concluded that this class of drugs are beneficial in the treatment of heart failure [132]. Dapagliflozin is a SGLT2 inhibitor drug with cardioprotective properties [129, 133]. Data from the DApagliflozin on renal outcomes and cardiovascular mortality in PAtients with Chronic Kidney Disease (DAPA-CKD) study suggested that dapagliflozin may be an efficient therapy for heart failure, though more complete analysis of data from this trial is required before any substantive conclusions can be made [129]. Additionally, the potential role of renin-angiotensin-aldosterone system blockade in halting heart failure development among diabetic patients has also been highlighted [120]. Angiotensin-converting enzyme inhibitors (ACE-I) are currently the first-line treatment for heart failure [134]. The BENEDICT (BErgamo NEphrologic DIabetes Complications Trial) and ADVANCE (Action in Diabetes and Vascular Disease-preterAx and diamicroN Controlled Evaluation) trial concluded that ACE-I help in delaying microalbuminuria onset in persons with T2DM, leading researchers to postulate that this may be an additional mechanism underlying the effectiveness of ACE-I in heart failure treatment, in addition to its vasodilatory action [135, 136].

### Albuminuria and Peripheral Arterial Disease

Peripheral arterial disease (PAD) is caused by large and small blood vessel damage resulting in ischaemia and necrosis of the extremities (typically the legs and feet). Diabetes and atherosclerosis are major causes of PAD, and the loss of circulation can lead to critical limb ischaemia and may require lower limb amputation. The potential link between albuminuria and PAD in patients living with diabetes has been investigated, with different studies providing conflicting evidence. A cross-sectional study performed among 1197 patients with T2DM showed that PAD was associated with albuminuria [137]. This was supported by the Multi-Ethnic Study of Atherosclerosis (MESA) study which showed that the risk of developing PAD was almost doubled in patients presenting with albuminuria [138]. Despite the positive correlation between albuminuria and PAD among diabetic subjects, the results from the National Health and Nutrition Examination Survey 1999–2004 showed that this association was also observed among non-diabetic subjects. Non-invasive methods to identify PAD, such as the use of Doppler ankle-brachial pressure index, are more practical for general population studies of the prevalence of PAD than gold standard angiography [139]. A number of pathological mechanisms have been suggested to contribute to the development of PAD among patients with albuminuria. In the diabetic milieu, endothelial dysfunction produces an excess of reactive oxygen species (ROS) which subsequently leads to an increase in platelet activity and vascular smooth muscle proliferation. This phenomenon is believed to play a significant role in the development and progression of PAD [140].

## Conclusion

Diabetes-associated complications are major contributors to patient morbidity and mortality, as well as to the global healthcare burden. Current and emerging treatments for diabetic vascular complications target a range of processes aimed at preserving vascular function and maintaining tissue homeostasis. Albuminuria has been identified to be a useful diagnostic marker of DN, indicative of damage to the glomerular filtration barrier. The hyperglycaemic state observed in diabetes results in aberrant cellular signalling causing damage to the kidneys, eyes, heart and other organs. The kidneys can be severely affected in diabetes, with vascular and tubular structures undergoing detrimental pathological changes as a result of hyperglycaemia which in due course leads to albuminuria. The damage to the glomerular capillaries can be considered to be representative of the widespread vascular damage evoked in the diabetic milieu.

**Fig. 2:**
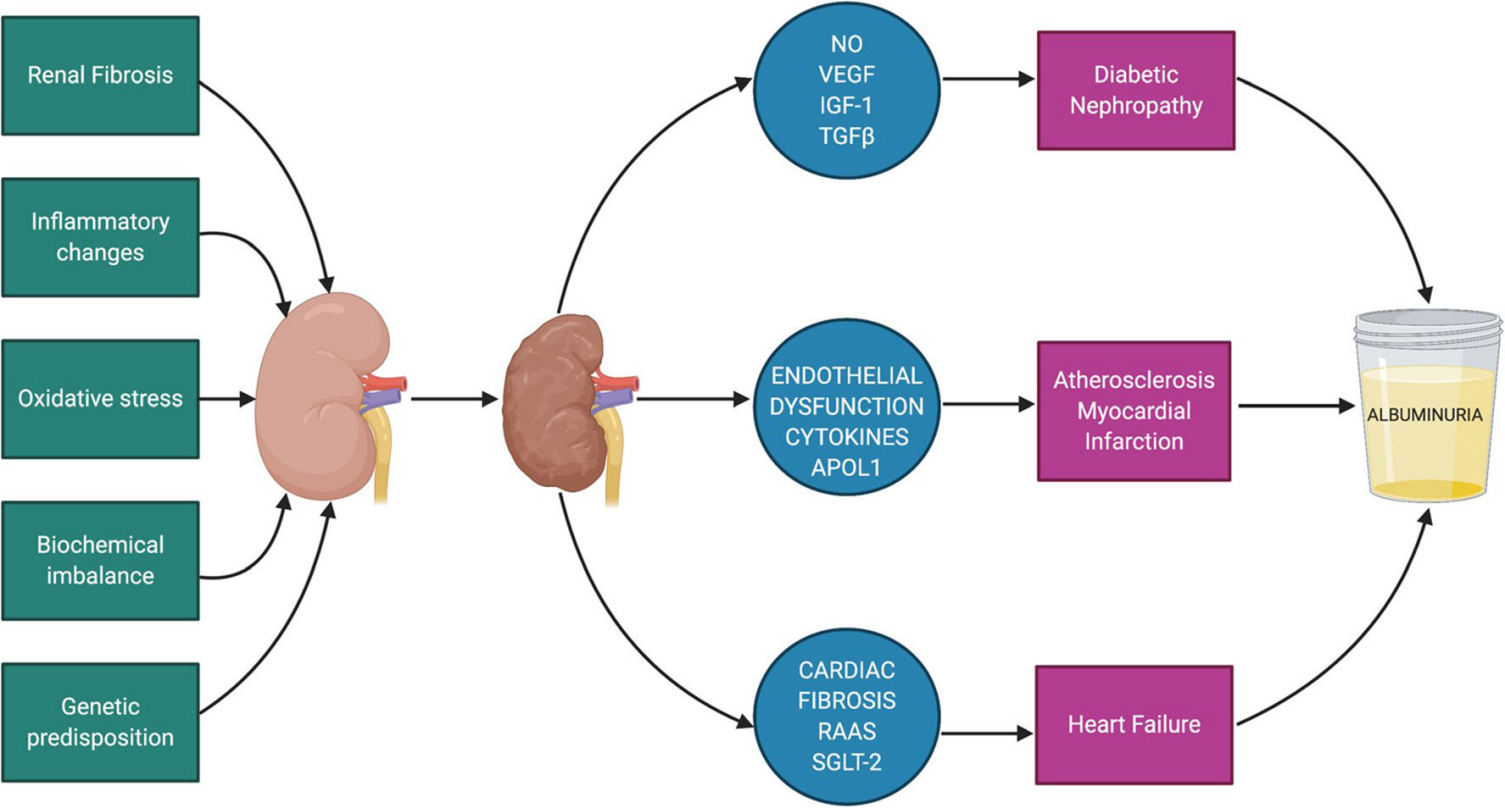
Summary of mechanisms leading to diabetic kidney disease and albuminuria

The increased CVD risk conferred by albuminuria has been well established in the literature. The pathogenic changes induced by albuminuria can accelerate the development of many cardiovascular conditions such as atherosclerosis, MI and heart failure. A summary of potential mechanisms of albuminuria as a result of micro- and macrovascular diabetic complications is shown in Fig. 2. Despite substantial evidence, the significance of albuminuria in diabetic complications has yet to be included in many clinical trials. The complex pathological processes involved have given researchers a wealth of biochemical pathways to study further. Continued and future support for pre-clinical and clinical studies into the significance of albuminuria will be fundamental in successfully translating animal studies into improved vascular outcomes for patients living with diabetes.
